# Impact of Heat Treatment on the Mechanical Properties and Fracture Morphology of Ti555211 Alloy

**DOI:** 10.3390/ma17143445

**Published:** 2024-07-12

**Authors:** Yushe Gao, Xiangyi Xue, Yuxuan Du, Xianghong Liu, Huixian Gao, Jianguo Wang, Junfeng Xu

**Affiliations:** 1 School of Materials Science and Engineering, Northwestern Polytechnical University, Xi’an 710072, China; 2 Western Superconducting Technologies Co., Ltd., Xi’an 710018, China

**Keywords:** Ti555211 titanium alloy, tensile strength, solid solution, annealing

## Abstract

Heat treatment is important for optimizing the strength performance and improving the toughness of titanium alloys. In this study, we investigated the impact of three heat treatment methods (β-annealing, double annealing, and solid-solution and aging treatment) on the mechanical properties and fracture morphology of Ti555211 titanium alloy. The results show that after β-annealing treatment, the alloy retains a high strength, while showing almost no ductility, and no yield strength. The alloy after double annealing has a high elongation rate (~54%) and lower strength. After solid-solution and aging heat treatment, the alloy was able to retain both high strength and a certain degree of ductility. The optimal heat-treatment process is solid-solution treatment at 820 °C/2 h and aging at 560 °C/12 h, which results in a maximum tensile strength of 1404 MPa and an elongation rate of 11%.

## 1. Introduction

Titanium alloys are very important aviation alloys due to their lightweight and high-strength properties [1]. However, they are also relevant in other technological sectors, for instance Koizumi et al. studied the application of titanium alloys to fixing dental prostheses [2]. Chirico et al. studied the effect of alloying elements on titanium hydride decomposition and a mechanism was proposed for explaining the dehydrogenation process [3]. Tedman-Jones et al. investigated titanium sponges as a possible common-denominator source of nuclei and found that the native nuclei populations in titanium alloys may consist of transient interstitial-based compounds and fragments of titanium [4].

The heat treatment of titanium alloys mainly includes annealing, solid-solution treatment, and aging [5,6,7]. Annealing can be divided into several forms based on the purpose of the heat treatment, including stress-relief annealing [8], complete annealing [9], double annealing [10], isothermal annealing, and dehydrogenation vacuum annealing [11]. The solid-solution treatment can be divided into two types according to the temperature: when it is carried out above the phase-transition temperature, it is abbreviated as β solid solution; when it is carried out below the phase-transition temperature, it is abbreviated as α + β solid solution [12,13]. For aging treatment, there are under aging, peak aging and over aging [14].

The relatively new Ti555211 titanium alloys are already widely used in the preparation of various metal components for aerospace spacecraft [15,16]. However, there have been only limited research reports so far. An et al. studied the deformation behavior of an isothermally compressed Ti555211 titanium alloy using an Arrhenius-type constitutive model and experimental data obtained from hot-compression tests; these tests were performed at temperatures and strain rates of 750–950 °C and 0.001–1 s^−1^, respectively [15]. Gao et al. investigated the high-temperature constitutive behavior of Ti555211 titanium alloy subjected to plastic deformation in the different phase regions. By comparing the constitutive equation of the alloy in the dual-phase zone with that of the alloy in the single-phase zone, they found that the deformation activation energy of the former is higher than that of the latter [16]. Zhen et al. studied the microstructure evolution of Ti555211 alloy during high-temperature deformation, and found that the content of the primary α phase decreases with the increase in deformation temperature. The effect of strain rate on microstructure variables of isothermally compressed Ti555211 alloy is mainly dependent on the deformation temperature [17]. Heat treatment can improve the comprehensive mechanical properties of materials, but reports about Ti555211 alloy heat treatment are still lacking.

To improve the comprehensive mechanical properties of Ti555211 titanium alloy, this study mainly focusses on the effects of three heat-treatment methods (β-annealing, double annealing, and solid-solution aging treatment) on the microstructure and properties of Ti555211 titanium alloy.

## 2. Experimental Methods

In the present work, Ti555211 titanium alloy was made in Western Superconducting Technologies Co., Ltd. (Xi’an, China). The (α + β)/β transition point is determined by the metallographic method (see Section 3.1). The test sample of Ti555211 alloy is the cylindrical bar with the size of Φ 350.

In this study, we adopt three methods of heat treatment. First is the β-annealing treatment, where the annealing temperature for Ti555211 titanium is decided by α + β/β phase-transition temperature. The phase-transition temperature of Ti555211 alloy is determined at 875–880 °C, so the annealing temperature is near 880 °C. The details for the β-annealing process are listed in Table 1.

The heat treatment process of double annealing mainly refers to TC18 (Ti-5Al-5Mo-5V-1Cr-1Fe) alloy, which should be selected at <880 °C. The metastable phase is reserved through solid solution and slow cooling, and then decomposed by aging to improve the strength of the material. The detail for double annealing is also listed in Table 1.

The heat treatment system of solid solution and aging is determined based on experience with other titanium alloy materials. The solid solution temperature is selected to be around 800 °C, and the aging temperature is selected to be 620 °C and listed together with the annealing time in Table 1.

The mechanical properties of the samples were tested after the heat treatment. The microstructure was observed by optical microscopy and scanning electron microscopy.

## 3. Results

### 3.1. Determination of Phase Transition Temperature

**Figure 1:**
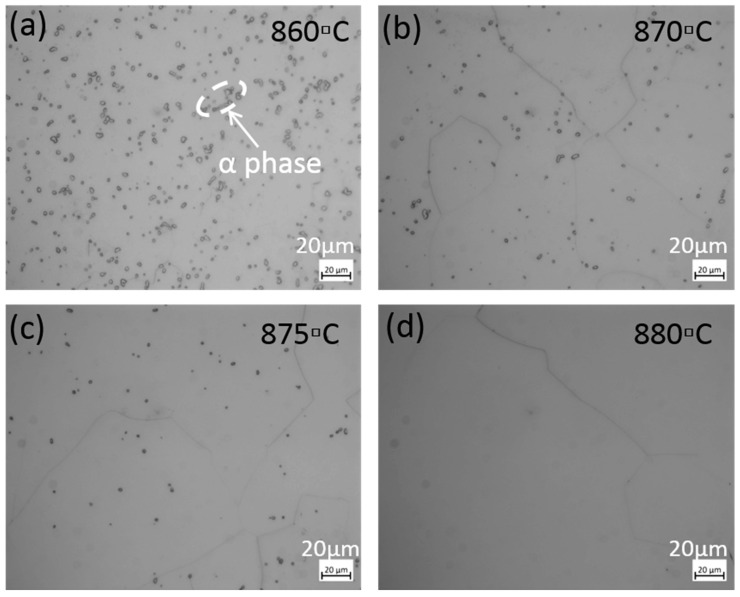
The phase transition temperature measured by the metallurgical method for Ti555211 titanium alloy: (**a**) 860 °C; (**b**) 870 °C; (**c**) 875 °C; (**d**) 880 °C.

**Figure 2:**
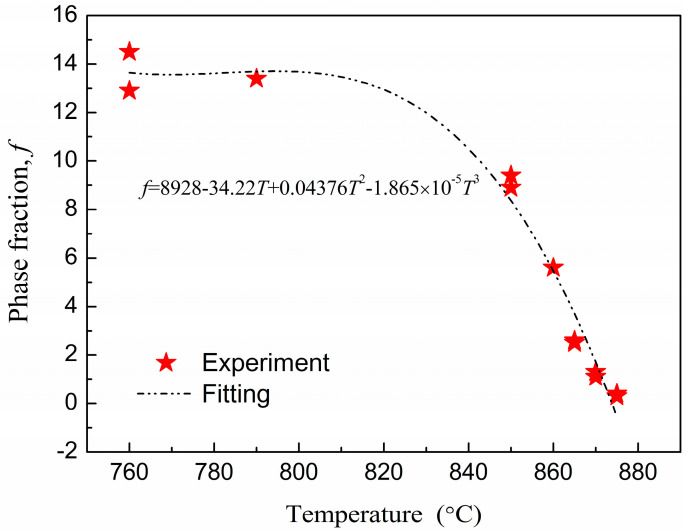
Trend of α phase fraction with temperature for Ti555211 titanium alloy. The broken line is an extrapolation using a polynomial function with parameters as indicated in the figure.

The temperature of α + β/β transition in titanium alloy, *T*_β_, has been defined as the lowest temperature at which all α-phase grains disappear from β matrix. To determine *T*_β_ for Ti555211 titanium alloy, the metallographic method was used to observe phase fractions of α + β and β phases in the microstructure. In accordance with the national standard of the People’s Republic of China GB/T 23605-2020 for the metallographic method, the main requirements are: (1) First of all, ensure that the temperature uniformity of the electric furnace used for heat treatment specimens is ≤3 °C; (2) The size of all specimens is guaranteed to be consistent, with a diameter of 10 mm ± 1 mm and a height of 15 mm ± 1 mm; (3) Metallographic assessment is assessed according to the standard (the test surface of the sample should be able to better represent the entire cross-sectional structure of the material. Considering the influence of microstructural fluctuations in different parts of the sample on the microstructure, at least five fields of view should be observed at the center and half radius of the sample, and a representative field of view should be selected to determine the content of phase A. The determination of phase content can be done using an image comparison method, image analysis software (Image J, https://www.statistical-analysis.top/ImageJ/Details.html?bd_vid=9848313351479648088, accessed on 9 May 2024), or calculation method, and the selected image magnification should be consistent). The specimen holding time is 35 min ± 5 min. The holding time was calculated from the time when the effective working area of the furnace reaches the set temperature. After treatment the sample is cooled by water quenching. The metallographic structure of Ti555211 titanium alloy samples after different temperature treatments are obtained as shown in Figure 1. The higher the temperature, the less α phase can be observed. Then, α-phase fractions corresponding to different temperatures are quantitatively measured as shown in Figure 2, with the trend of α-phase fraction and temperature variation extrapolated using a simple polynomial function.

From the extension line of the trend of phase-fraction variation, one can determine that the α + β/β phase-transition temperature is between 875 °C and 880 °C for Ti555211 titanium alloy ingots. The microstructure analysis confirms that the α phase completely disappears under the 880 °C treatment (Figure 1d).

### 3.2. Microstructure and Performance after β-Annealing

**Figure 3:**
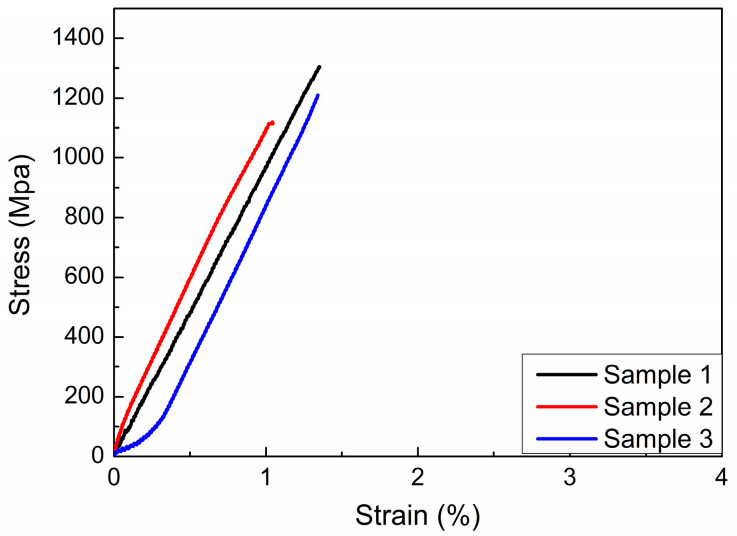
The tensile properties of β-annealing treatment samples.

**Figure 4:**
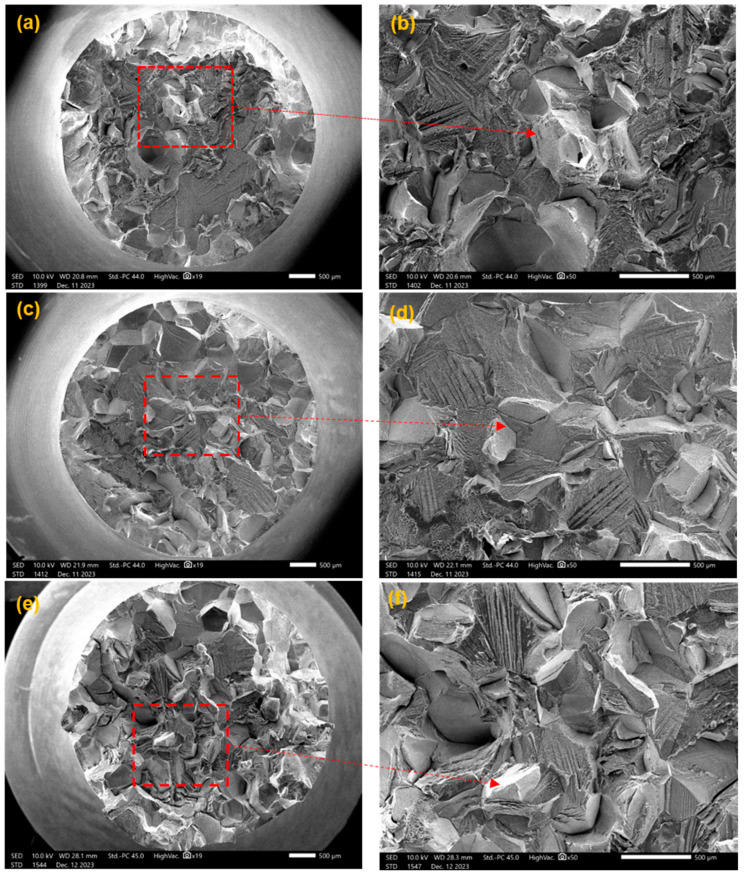
The tensile fracture morphology of the sample with β-annealing: (**a**,**b**) Sample 1, 870 °C/8 h AC; (**c**,**d**) Sample 2, 880 °C/8 h AC; (**e**,**f**) Sample 3, 890 °C/8 h AC.

For general titanium alloys, β-annealing treatment can change the solid solubility of certain phases to improve the strength of materials [18]. Figure 3 shows the tensile curve for samples that have undergone different β-annealing treatments (870 °C/8 h, 880 °C/8 h, 890 °C/8 h, See Table 1). The tensile strength of all three samples exceeded 1100 MPa, but brittle fractures occurred before reaching the yield stage. Figure 4 shows the fracture morphology and local magnified image of the β-annealed sample. It can be seen that the fracture surface exhibits brittle cleavage fracture, with local smoothness and the appearance of grain-shaped crystal planes. This indicates that the tensile process will suddenly undergo brittle fracturing without yield strength, and the shrinkage of the fracture surface can be ignored.

Table 2 shows the mechanical properties of the β-annealing treatment sample of Ti555211 titanium alloy. It shows that the impact toughness is relatively high, but the strength of the material has significantly decreased. The sample treated at 880 °C has the lowest strength (R_m_) and impact energy (A_K_). As the microstructure transformed from a two-phase zone to a single-phase zone, the material plasticity in the single-phase zone is poor, and the aging strengthening effect is weakened. It indicates that under β-annealing treatment, the strength adjustment range is relatively small. At the same time, the large furnace mass hinders rapid cooling of the sample. Within a certain range, increasing the cooling rate of the furnace can appropriately increase the strength of the material. Therefore, β-annealing has fewer heat-treatment advantages and cannot fully result in the superior strength and toughness of Ti555211 titanium alloy.

### 3.3. Microstructure and Performance after Double Annealing Treatment

**Figure 5:**
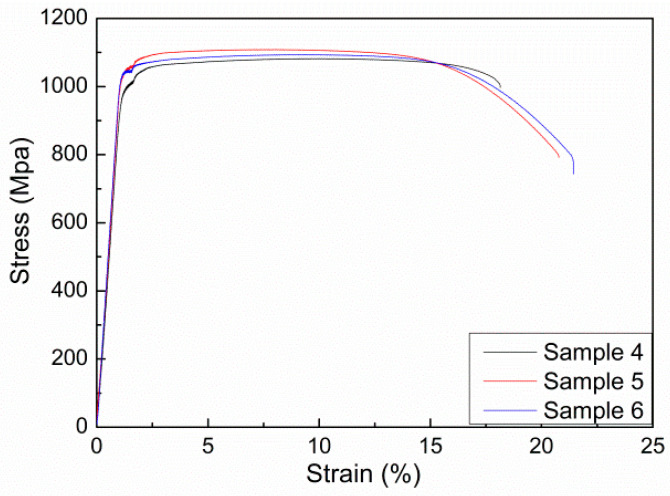
Tensile curve of the sample after double annealing.

Double annealing can generally improve the ductility of materials. The annealing process of Ti555211 titanium alloy has been shown in Table 1. Figure 5 shows the tensile curve of the sample after double annealing. Although the strength of the sample after double annealing is not as high, the elongation is substantially increased, indicating excellent ductility.

Table 3 shows the properties of Ti555211 titanium alloy after double-annealing treatment. The highest strength is only 1109 MPa. The highest elongation and cross-sectional shrinkage rate are 54% and 20.5%. Therefore, the comprehensive performance advantage of the sample after double annealing is relatively low. The strength is not so high that it can only be used for cases with strength requirements below 1100 MPa.

**Figure 6:**
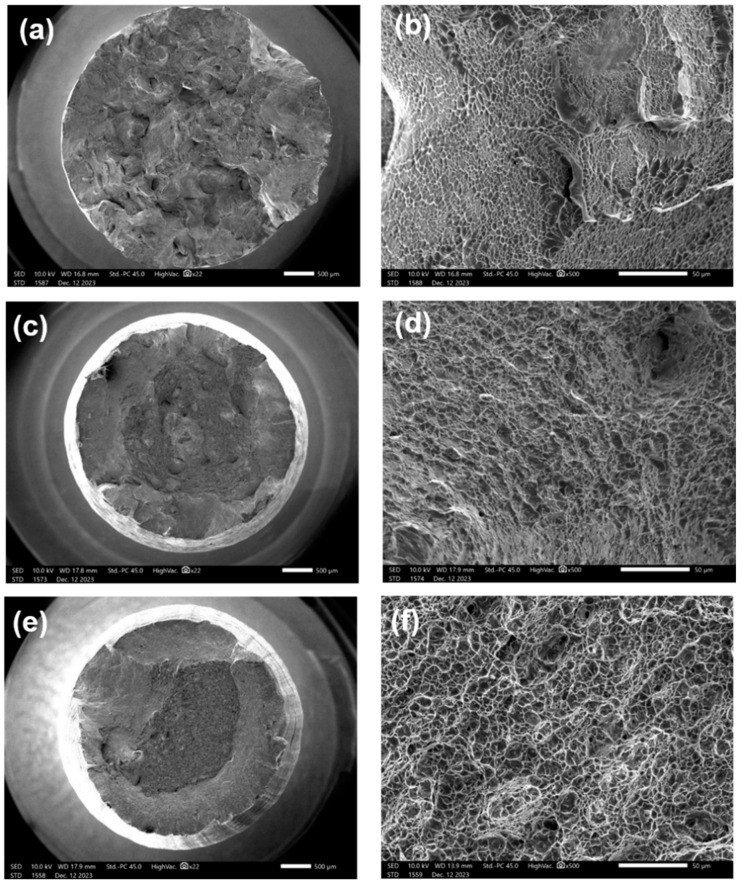
Fracture morphology of double-annealing sample: (**a**,**b**) Sample 4, 880 °C/1.5 h and 760 °C/8 h, AC and 620 °C/8 h, AC; (**c**,**d**) Sample 5, 860 °C/1.5 h and 760 °C/8 h, AC and 620 °C/8 h, AC; (**e**,**f**) Sample 6, 840 °C/1.5 h and 760 °C/8 h, AC and 620 °C/8 h, AC.

Figure 6 shows the tensile fracture morphology after double-annealing treatment. It can be seen that the fracture surface appears as many mesh-like patterns, containing many ductile dimples, especially for the samples annealed at 860 °C and 840 °C. Radial ridge-like patterns can also be observed, with obviously brittle fracture characteristics. However, significant shrinkage deformation occurred at the fracture surface, indicating strong plastic deformation ability.

**Figure 7:**
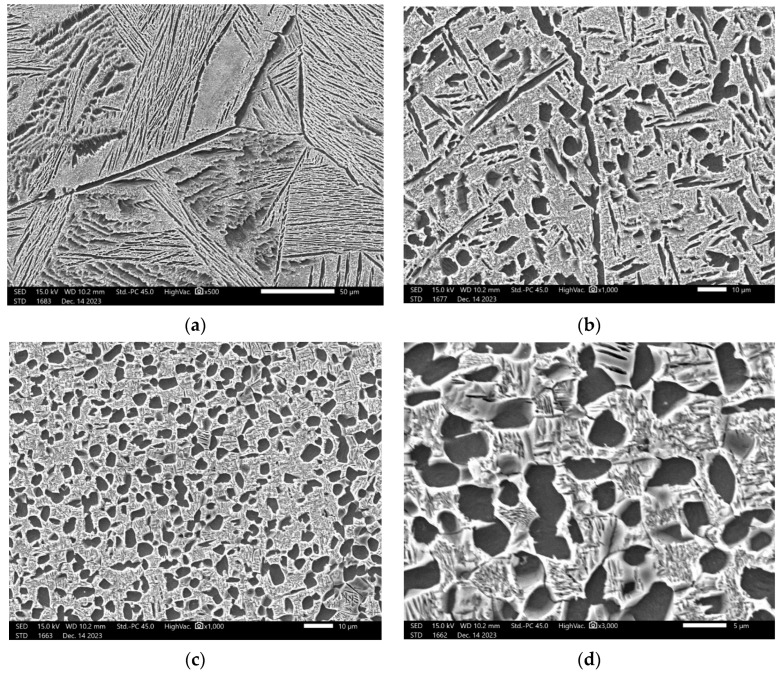
Microstructure of the sample after double annealing: (**a**) Sample 4, 880 °C/1.5 h cooling in furnace to 760 °C/8 h, AC and 620 °C/8 h, AC; (**b**) Sample 5, 860 °C/1.5 h cooling in furnace 760 °C/8 h, AC and 620 °C/8 h, AC; (**c**) Sample 6, 840 °C/1.5 h cooling in furnace 760 °C/8 h, AC and 620 °C/8 h, AC; (**d**) Sample 6, 840 °C/1.5 h cooling in furnace 760 °C/8 h, AC and 620 °C/8 h, AC.

Figure 7 shows the microstructure of Ti555211 titanium alloy after double-annealing treatment. It can be seen that under the condition of second annealing at 760 °C, reducing the first annealing temperature from 880 °C to 840 °C will result in the α phase gradually changing from needle-like to equiaxed or circular. This means that the plastic deformation ability of the material increases, because under general conditions, the plastic deformation ability of equiaxed grain materials is much better than that of columnar structure, and even better than that of a needle-like Widmannstatten structure. The experimental results data in Table 3 also prove this. They show that the cross-sectional shrinkage and elongation of the samples treated at 880 °C are 17% and 26%, respectively, while the cross-sectional shrinkage and elongation of the samples treated at 840 °C can reach 20% and 56%, respectively.

### 3.4. Microstructure and Performance after Solid Solution and Aging Treatment

**Figure 8:**
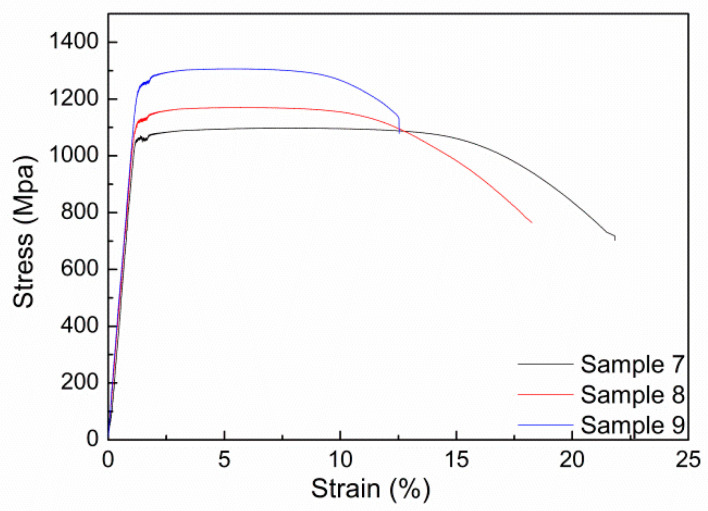
Tensile curve of the samples after solid-solution and aging treatment.

The solid-solution and aging heat treatment process can often simultaneously improve the strength and ductility of materials. According to the process in Table 1, The sample was subjected to solid-solution and aging treatment, and after that tensile testing was performed. The obtained tensile curve is shown in Figure 8. The summary of its tensile performance is shown in Table 4.

Other mechanical properties of the sample after solid-solution and aging treatment are also listed in Table 4. It can be seen that, as the solid-solution temperature increases, the range of tensile strength of the sample can change from 1091 MPa to 1306 MPa, and the elongation can change from 26% to 57%. From Table 4, it can also be seen that the impact energy of these samples has decreased compared to the double-annealed samples. This means that solid-solution treatment has a greater effect on improving material strength and a smaller impact on ductility.

**Figure 9:**
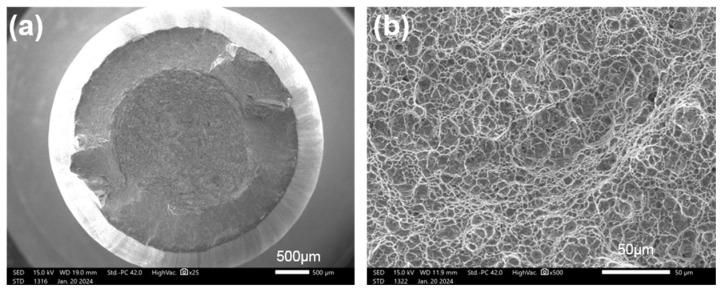

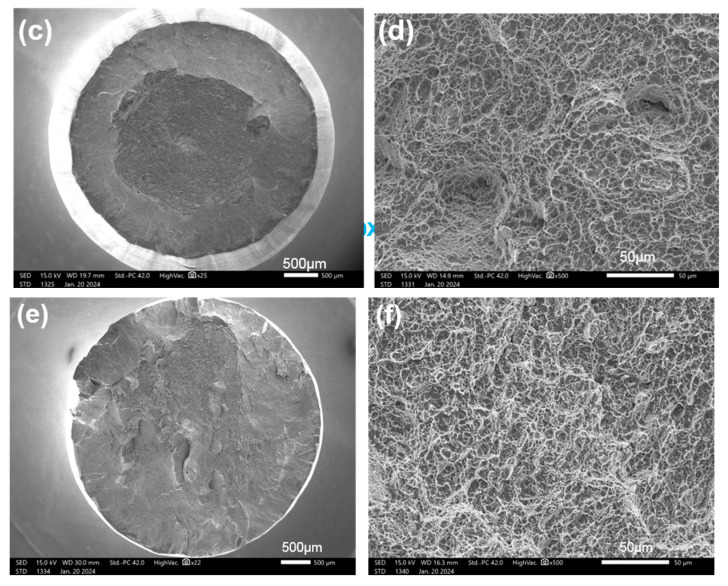
Fracture morphology of solid-solution and aging treatment sample: (**a**,**b**) Sample 7, 760 °C/2 h AC and 620 °C/8 h AC; (**c**,**d**) Sample 8, 800 °C/2 h AC and 620 °C/8 h AC; (**e**,**f**) Sample 9, 840 °C/2 h AC and 620 °C/8 h AC.

Figure 9 shows the fracture structure after solid-solution and aging treatment. It can be seen that there are many small ductile dimples on the fracture surface of the sample, which is a characteristic of detailed ductile fracture. However, there are still slight differences in the number and size of ductile dimples among these samples, indicating a certain difference in strength and toughness of the samples. By comparing the tensile strength listed in Table 4, it can be observed that the dimples in Figure 9f are the smallest, most numerous, and have the highest strength.

**Figure 10:**
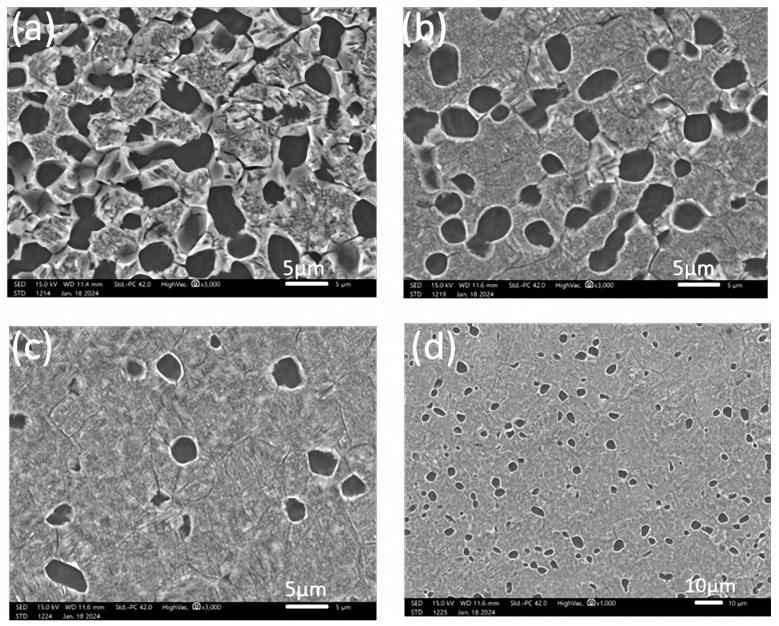
Effect of solid solution and aging treatment on the microstructure of Ti555211 alloy: (**a**) Sample 7, 760 °C/2 h AC and 620 °C/8 h AC; (**b**) Sample 8, 800 °C/2 h AC and 620 °C/8 h AC; (**c**) Sample 9, 840 °C/2 h AC and 620 °C/8 h AC (high magnification); (**d**) Sample 9, 840 °C/2 h AC and 620 °C/8 h AC (low magnification).

The microstructures of Ti555211 titanium alloy after solid-solution and aging treatments of 760 °C/2 h AC and 620 °C/8 h AC, 800 °C/2 h AC and 620 °C/8 h AC, and 840 °C/2 h AC and 620 °C/8 h AC are shown in Figure 10. It can be seen that the microstructure of Ti555211 titanium alloy in the two-phase zone is a primary α_p_ phase, which is equiaxed or to a low extent rod-shaped, dispersed, and distributed in the β substrate (Figure 10a–c). The β matrix continuously precipitates fine and layered phase α_s_ during the aging process. The fraction of primary α_p_ phase decreases with the increase of solid-solution temperature. After solid-solution treatment at 760 °C and 800 °C, the fraction of primary α_p_ phase is basically 19.2~31.8%, and the size of primary α_p_ phase is 3.04~3.28 μm. The fraction of primary α_p_ phase significantly decreased with the increase of the solid solution temperature. The size of the primary α_p_ phase also decreased with the increasing solid-solution temperature. The primary α_p_ phase is 7.56% if the solid-solution temperature is 840 °C. Therefore, below the phase transition point, as the solid solution temperature increases, the content of α phase decreases and the size decreases.

Due to the fact that solid solution and aging can achieve better strength performance, after repeated experiments on the sample, Sample 10 with higher strength and a certain elongation rate was finally obtained. Its maximum tensile strength can reach 1404 MPa, and the elongation rate remains at 11%. Its heat treatment process is 820 °C/2 h, AC and 560 °C/12 h.

## 4. Discussion

The experiment above indicates that three heat-treatment processes (β-annealing treatment, double annealing, and solid-solution and aging treatment) are applied to the titanium alloy, all having an impact on mechanical properties; however, the sample after β-annealing has almost no yield strength, indicating that the material becomes brittle. After double-annealing treatment, the alloy ductility has been improved to a certain extent, but the strength value is not high enough to meet the requirements for use. Compared to others, solid solution and aging treatment can significantly improve strength and plastic properties.

Analyzing the reasons from a microscopic perspective, we find that needle-like α phase appears near grain boundaries after β-annealing treatment. In β grains, some α secondary grains also precipitate, presenting Widmanstatten structure characteristics, resulting in brittle properties of the sample. Both Widmanstatten structures and needle-like structures lead to high stress energy stored at the tip of the needle, and the stress concentration reduces the strength of microstructure, thereby increasing the fragility of the Ti555211 alloy.

The microstructure of the Ti555211 titanium alloy rod after double annealing is an equiaxed crystal structure (Figure 7). Primary α_p_ phase is spheroidization. However, the microstructure after double annealing and solid solution and aging in primary and secondary phase α_s_ has some difference in the size. After double annealing (Figure 7), the grain size of primary α_p_ phase is relatively large. The elongated second α_s_ phase is scattered in β matrix, and some of them have been coarsened. After solid solution and aging (Figure 10), the grain size of the primary α_p_ phase is smaller than that of double annealing, and secondary annealing α_s_ phase size is also finer, distributed in a needle-like manner and intertwined with each other. This kind of microstructure has an obvious strengthening effect on the titanium alloy matrix. At the same time, due to the secondary α_s_ phase, it is so small that it reduces the capacity for crack propagation, which is reflected in the low impact toughness of the performance test results. 

The microstructure from β-annealing is mainly composed of a coarse layered structure, which is conducive to hindering crack propagation. Whereas under the β-phase process a series of recovery and recrystallization occurs during the heat-treatment process, leading to decreased ductility, and a decrease in the strengthening effect of dislocations. In this state, the strength and ductility are relatively poor. The solid-solution and aging microstructure is mainly composed of a small amount of equiaxed primary α_p_ phase and neatly arranged layers of secondary α_s_ phase. The spheroidization of the primary α_p_ phase improves the ductility of the alloy, and the secondary precipitation of the α_s_ phase improves the strength and toughness of the alloy. Under the action of two conditions, the toughness advantage of Ti555211 alloy is exerted.

Based on the above results, the solid solution and aging treatment method leads to the greatest improvement on the performance of Ti555211 alloy. In addition, from Table 1, Table 2, Table 3 and Table 4, we find that the lower the heat-treatment temperature, the better the strength performance, the longer the heat-treatment time and the higher the elongation.

Recently, Liu et al. have report that after heat treatment for Ti–6Al–4V titanium alloy, the optimal tensile strength, yield strength, and elongation were 999, 919 MPa and 10.4%, respectively [19]. Lu et al. reported mechanical properties of shock-modified titanium alloy, the yield strength, ultimate tensile strength, and fracture strain derived from stress–strain curves are 1.02–1.18 GPa, 1.23–1.41 Gpa, and 7.4–10.9%, respectively [20]. Compared with these alloys, the strength properties and ductility properties of Ti555211 alloy (Sample 10) are better.

## 5. Conclusions

The transition temperature of (α + β)/β phase was measured by the metallographic method as 875–880 °C for Ti555211 alloy. According to the (α + β)/β phase transition temperature, the β-annealing treatment was set as 870–890 °C; the double-annealing treatment was set as 880–840 °C; the solid-solution and aging treatment was set as 840–760 °C. The results show that after β-annealing, the Ti555211 alloy sample has higher tensile strength (~1315 MPa) and lower elongation, but no yield strength. After double-annealing treatment, the sample exhibits high ductility (~54%) and low strength. After solid-solution and aging treatment, the sample can achieve a maximum tensile strength of 1404 MPa and an elongation of 11%, which meets the requirements of high strength and a certain degree of ductility. In addition, from the overall heat-treatment process and performance results, it was found that the lower the heat treatment temperature, the better the strength performance, and the longer the heat treatment time and the higher the elongation.

## Figures and Tables

**Table 1:** β-annealing, double annealing, and solid solution and aging heat treatment processes of Ti555211 titanium alloy. AC is air cooling.

Heat Treatment Method	Samples	Processes Details
β-annealing	Sample 1	870 °C/8 h, AC + 620 °C/8 h, AC
	Sample 2	880 °C/8 h, AC + 620 °C/8 h, AC
	Sample 3	890 °C/8 h, AC + 620 °C/8 h, AC
Double annealing	Sample 4	880 °C/1.5 h + 760 °C/8 h, AC + 620 °C/8 h, AC
	Sample 5	860 °C/1.5 h + 760 °C/8 h, AC + 620 °C/8 h, AC
	Sample 6	840 °C/1.5 h + 760 °C/8 h, AC + 620 °C/8 h, AC
Solid solution and aging	Sample 7	760 °C/8 h, AC + 620 °C/8 h, AC
	Sample 8	800 °C/8 h, AC + 620 °C/8 h, AC
	Sample 9	840 °C/8 h, AC + 620 °C/8 h, AC
	Sample 10	820 °C/2 h, AC + 560 °C/12 h, AC

**Table 2:** Mechanical Properties of Ti555211 Alloy after β-Annealing Treatment.

Heat Treatment Processes	Tensile Strength, R_m_/MPa	Impact Energy, A_K_/J
Sample 1 (870 °C)	1315	8.16
Sample 2 (880 °C)	1120	7.84
Sample 3 (890 °C)	1224	8.88

**Table 3:** Mechanical properties of Ti555211 alloy after double-annealing treatment. *R*_m_ is the tensile strength; *R*_p0.2_/ is yield strength; *A*/% is section shrinkage; *Z*/% is elongation.

Heat-Treatment Processes	*R*_m_/MPa	*R*_p0.2_/MPa	*A*/%	*Z*/%	*A*_K_/J
Sample 4 (880 °C, 760 °C)	1082	990	17	26	37.84
Sample 5 (860 °C, 760 °C)	1109	1044	20	54	37.12
Sample 6 (840 °C, 760 °C)	1094	1039	20.5	52	9.04

**Table 4:** Mechanical properties of Ti555211 alloy after solid-solution and aging treatment.

Heat Treatment Processes	*R*_m_/MPa	*R*_p0.2_/MPa	*A*/%	*Z/*%	*A_K_*/J
Sample 7 (760 °C)	1091	1057	21.5	57	30
Sample 8 (800 °C)	1170	1123	17.5	57	21.4
Sample 9 (840 °C)	1306	1246	11.5	26	22.5
Sample 10 (840 °C)	1404	1346	11	29	25.2

## Data Availability

The original contributions presented in the study are included in the article, further inquiries can be directed to the corresponding authors.
